# A qualitative study of the anticipated barriers and facilitators to the implementation of a lifestyle intervention in the dutch construction industry

**DOI:** 10.1186/1471-2458-14-1317

**Published:** 2014-12-23

**Authors:** S C Tonnon, K I Proper, H P van der Ploeg, M J Westerman, E Sijbesma, A J van der Beek

**Affiliations:** Department of Public and Occupational Health, EMGO+ Institute of Health and Care Research, VU University Medical Center, Amsterdam, The Netherlands; Department of Methodology and Statistics, EMGO+ Institute of Health and Care Research, VU University, Amsterdam, The Netherlands

**Keywords:** Cardiovascular risk, Primary prevention, Occupational, Lifestyle intervention, Implementation, Barrier, Qualitative

## Abstract

**Background:**

Lifestyle interventions have proven effective for lowering a cardiovascular risk profile by improving lifestyle behaviors, blood glucose and blood cholesterol levels. However, implementation of lifestyle interventions is often met with barriers. This qualitative study sought to determine anticipated barriers and facilitators to the nationwide implementation of an effective lifestyle intervention in the construction industry in the Netherlands.

**Methods:**

Prior to implementation, focus groups were held with 8 lifestyle counselors and semi-structured interviews with 20 employees of the construction industry, 4 occupational physicians, 4 medical assistants, and 1 manager of an occupational health service. The transcripts were coded by two coders and analyzed by constant comparison.

**Results:**

Hypothetical employee willingness to sign up for the intervention was facilitated by a high level of perceived risk, perceived added value of the intervention, and perceived social support. It was hampered by a preference for independence and perceived interference with their work. All professionals named a lack of time as an anticipated barrier to implementation. Lifestyle counselors suggested several strategies to improve the proficiency of their counseling technique, such as training in small groups and a continuous stream of employee referrals. Occupational physicians thought they would be hampered in screening employees and referring them to a lifestyle counselor by the perception that addressing employee lifestyles was not their task, and by a counter-productive relationship with other stakeholders. The manager addressed financial incentives and a good intervention fit with the current approach of the OHS.

**Conclusion:**

The findings suggest that employees can be motivated to sign up for a lifestyle intervention by tailoring the implementation strategy to various subgroups within the target group. Occupational physicians can be motivated to refer employees for the intervention by making a referral personally and professionally rewarding.

**Electronic supplementary material:**

The online version of this article (doi:10.1186/1471-2458-14-1317) contains supplementary material, which is available to authorized users.

## Background

Cardiovascular disease (CVD) is the leading cause of death worldwide and accounts for 12% of disability adjusted life years among non-communicable diseases [1, 2]. Approximately 80% of coronary heart disease and cerebrovascular disease can be attributed to behavioral risk factors, such as smoking, physical activity and dietary intake [3]. Even small sustained lifestyle changes lead to significant gains at an individual and population level [4]. Reviews show that lifestyle interventions can effectively reduce CVD risk when they are multi-factorial, include an environmental component, target a high-risk group, and when there is regular contact with the individual [5–9].

Employees of the Dutch construction industry show elevated scores on several CVD risk factors in comparison with the adult Dutch male population, such as a higher prevalence of obesity (17% versus 9%), smoking (32% versus 29%) and physical inactivity (53% versus 41%) [10, 11]. To reduce CVD risk among employees in the construction industry, the Health Under Construction (HUC) intervention was developed in 2006-2010 [12]. This intervention seeks to achieve long-term behavioral change regarding a healthy diet and a physically active lifestyle through individual counseling [13, 14]. During a periodic medical examination (PME) at an occupational health service, occupational physicians (OP) screen employees for an elevated CVD risk and refer the risk group to a lifestyle counselor. Participants receive a minimum of two face-to-face consultations and three phone consultations with a counselor, who is trained in motivational interviewing techniques. A randomized controlled trial demonstrated positive effects of the HUC intervention on snack and fruit intake and body weight at six-months, as well as on snack intake, body weight, HDL-cholesterol and HbA1c at 12 months [15, 16]. Based on these results, the institute Arbouw, which coordinates occupational health care in the Dutch construction industry, decided to implement the HUC intervention at a national level.

Before implementation, the intervention was adjusted according to the findings of the earlier trial. First, older employees were more likely to sign up for the intervention than the younger ones and the intervention had a larger effect on their systolic blood pressure, HDL-cholesterol and physical activity [15, 16]. Therefore, the target group for the current study was defined as employees who were 40 years old or more [17]. Second, during the trial, only 20% of the target group signed up for the intervention [18]. In order to increase the reach of the intervention, the screening instrument was made available as an online tool and integrated into the administration system of the occupational health services (OHS) where possible. Furthermore, the process evaluation of the HUC intervention showed that the intervention could be improved by increasing the counselors’ proficiency in motivational interviewing [18]. To this end, the counselor training was expanded. Finally, since treatment for smoking cessation had recently become available in the Netherlands via the general practitioner, the smoking cessation part of the HUC intervention was not implemented.

Although the process evaluation alongside trial indicated several ways to improve the implementation of the intervention, a comprehensive assessment of the barriers and facilitators to the implementation was still lacking. An a priori assessment of the barriers and facilitators makes it possible to direct implementation strategies at these factors [19]. Implementation research shows that there are numerous barriers to the implementation of innovations in health care settings. For example, primary care physicians fail to screen, advise, or refer their patients for CVD risk because of a lack of time and a lack of services to refer to, because they doubt the usefulness of screening instruments, participation fees are not reimbursed to patients, or because they feel the target group is not motivated to participate in the intervention [20–24]. Potential participants fail to sign up for lifestyle interventions because of work commitments, participation fees, lack of transport, or misconceptions about their health status and about appropriate measures to address the problem [25, 26].

The current study provides new insights, because the implementation process under study takes place in a real-life setting. During the trial, the implementation process was coordinated by a research team. During the current study, coordination was taken over by the OHS, thus making the results more relevant for implementation processes outside a controlled setting. Furthermore, the process evaluation had focused exclusively on the role of the counselors and the employees, while during the present study, interviews were held with all key stakeholders of the implementation process, namely employees, OPs, medical assistants, counselors, and an OHS manager. The goal of this qualitative study was to gain insight into the barriers and facilitators to the implementation of a lifestyle intervention in the Dutch construction sector.

## Methods

### Study design

This study had a qualitative, explorative design and was based on focus groups and semi-structured interviews. In order to obtain in-depth information on the factors that influence the willingness and ability to participate and implement a lifestyle intervention in this specific context, a qualitative design was chosen. Focus groups were generally the preferred method because this approach allows for interaction among respondents and generates rich data [27]. However, in the context of the present study, participation in a focus group meant that the respondents would have to travel, thus risking a low response. The counselors had already participated in the earlier intervention trial, which made recruiting them easier and a focus group feasible. The OPs, medical assistants, and OHS manager were considered to be less motivated to participate in the current study and so they were interviewed individually. The Medical Ethics Committee of the VU University Medical Center decided that it was not necessary to seek ethical approval to conduct this study.

### Intervention and context

In the Netherlands, occupational health in the construction sector is financed and coordinated by the national institute Arbouw. This non-profit institute represents construction industry employers and employees and aims to improve working conditions and reduce sickness-related absence in the sector. Employers in the construction sector pay fees to Arbouw, which Arbouw uses to develop and implement health and safety measures. One of those measures is the periodic medical examination (PME). Every employee in the construction sector has the right, but is not obliged, to participate in one PME every two to four years. At the time of the present study, Arbouw had contracted with 25 commercial OHS to perform these PMEs. The OHS employ OPs, medical assistants and other professionals specialized in occupational health. One of the OHS major task is preventing sickness-related absence and facilitating employees’ return to work. OHS professionals perform PMEs and follow-up interventions. OHS professionals work with employees of several industries, but since the construction industry has one of the largest work forces in the Netherlands, some professionals specialize in the construction sector and have worked with these employees for many years. Arbouw provides the OHS with guidelines on how to perform a PME and monitors their performance. During a PME, the designated OPs and medical assistants perform biomedical assessments, collect and review employee health questionnaire data, and, if necessary, refer employees for follow-up tests or treatment to general practitioners (GPs), medical specialists, or occupational health consultants, such as lifestyle counselors.

Implementation of the HUC intervention requires that OPs systematically screen employees for an elevated CVD risk and refer those at risk to a trained lifestyle counselor. Prior to implementation, each OHS selected one or more of their employees, preferably with counseling experience, and assigned them to the four-day HUC training organized by Arbouw. The counselors’ professional background ranged from medical assistant to social worker. The training consisted of four modules: 1) motivational interviewing, 2) the relationship between physical activity, diet, and CVD risk, and 3) the HUC intervention protocol, and 4) data collection for the process evaluation of the national implementation.

When an employee was referred to a lifestyle counselor, the employee received 2–3 face-to-face consultations and 3–4 phone consultations. The face-to-face consultations took place at the OHS, the participant’s home, or the participant’s work place. Participants chose whether they wanted to change their diet, or increase their physical activity, and how they wanted to achieve those goals. The intervention was thus tailored to the participant’s needs and preferences. The counselor provided guidance on achieving these lifestyle changes if the participant so desired. Participation fees were covered by Arbouw.

### Theoretical framework

Fleuren, Paulussen and Wiefferink’s theoretical framework was used for this study [28]. The model was chosen because it was developed to analyze implementation processes in large health care organization as opposed to implementation by individual health professionals. The framework is based on the Theory of Planned Behavior and Social Cognitive Theory [29, 30]. Based on this framework, implementation processes pass through the stages dissemination, adoption, implementation, and continuation. Barriers and facilitators determine whether an innovation passes from one stage to the other. Fleuren et al. performed a literature review, that identified 50 determinants of implementation, which fall into one of the following five categories. 1) The socio-political level includes factors that relate to the larger political context, such as national laws, and factors related to patient characteristics, such as the patient’s beliefs about the innovation. 2) The organizational level is comprised of factors related to the organization that the health professional works for, such as staff capacity or available expertise. 3) The level of the health professional refers to the characteristics of the implementing professional. For example, the professional’s skills needed for implementation, as well as factors that directly affect the professional, such as support from colleagues. 4) The innovation level includes the characteristics of the innovation, such as the observability of the innovation or whether or not the innovation is appealing to use. 5) The facilities’ level includes factors such as financial resources or manuals. The topic lists were based on the research question. The concepts in the framework served as sensitizing concepts during the coding of the data. In a later step in the analysis, the data was structured according to the framework.

### Data collection

At the time of the data collection, there were 25 OHS providing services to the Dutch construction industry. Two of the 25 organizations managed about 80% of the service demand of the industry, thus they were the most relevant settings for the present study. These two OHS had also participated in the earlier trial of HUC intervention in 2006–2010. The professionals and the employees for the current qualitative study were recruited through these two OHS.

#### Employees

Employees were recruited for the current study by consecutive sampling. In order to make the interview setting comparable to the implementation setting, the employees were interviewed immediately after their PME. Employees have the right to take paid leave for their PME, therefore the PME usually takes place during the employees’ work time. It was not possible to select interview respondents according to their CVD risk level; the OPs who recruited employees for the interviews did not consent to providing information about the employees’ CVD risk assessment due to privacy issues. Consequently, respondents were recruited regardless of their CVD risk. During the PME, the OP asked those employees aged 40 years or older whether or not they wanted to participate in an interview for the current study. All participants gave oral consent for participation to the OP.

To introduce the subject of the interview, the interviewer asked the employee to imagine that the OP had just told them they had an elevated CVD risk, had advised them to sign up for a lifestyle intervention, and had given him or her a brochure. The interviewer read the brochure in question to the employee, which contained a description of the intervention. Employees were then asked whether they wanted to sign up for the intervention, and which factors had a role in their decision. In order to stimulate the employees to elaborate on their response, the employees were then asked to react to statements, that were read aloud by the interviewer and presented to the employee on cue cards (see Additional file 1). The statements operationalized barriers and facilitators that were derived from the interviews with the professionals who were included in the present study, as well as interviews with employees in the construction industry that had been held in 2006 in preparation for designing the HUC intervention [31].

The duration of the interviews ranged from 12–36 minutes. On average, interviews lasted 20 minutes. Table 1 summarizes the method, content, and duration of the interviews.

**Table 1:** **Method, content, and duration of the interviews per respondent group**

Respondent group	Method	Content	Average duration
Employee	individual interview	Instruction: "Let us suppose the OP just told you that you have an elevated CVD risk. He recommended you sign up for a lifestyle intervention and gave you the following brochure". Interviewer reads the brochure to the employee.	20 min
		• Open question: "Would you sign up for the intervention? Why would you/wouldn’t you?"	
		• Statements: The interviewer reads statements from cue cards to the employee and asks whether the factor in question had a role in his decision on signing up for the intervention.	
Occupational Physician	individual interview	Instruction: The interviewer gives a written description of the intervention and implementation plan to the OP.	60 min
		Questions:	
		• What is your current approach to CVD risk management and the promotion of a healthy lifestyle among employees of the construction industry?	
		• Which changes does the implementation of the intervention Health under Construction imply for the way you work?	
		• How would you go about implementing those changes?	
Medical Assistant	individual interview	Instruction: the interviewer gives a written description of the intervention and implementation plan to the medical assistant.	25 min
		Questions:	
		• How do you currently go about performing a PME for employees in the construction industry?	
		• Which changes does the implementation of the intervention Health under Construction imply for the way you work?	
		• How would you go about implementing those changes?	
Manager	individual interview	Instruction: the interviewer gives a written description of the intervention and implementation plan to the medical assistant.	90 min
		Questions:	
		• What is the current approach of your OHS to CVD risk management and the promotion of a healthy lifestyle among employees in the construction industry?	
		• Which changes does the implementation of the intervention Health under Construction imply for the way this OHS works?	
		How would you go about implementing those changes?	
Counselor	focus group	General questions to facilitate recall:	120 min
		• How did you go about performing the counseling for Health under Construction?	
		• What were useful elements of the intervention?	
		Questions: The process evaluation during the trial of Health under Construction showed that counselors found it difficult to	
		1) schedule five to seven consultations within a period of six months,	
		2) apply the motivational interviewing technique	
		3) motivate participants to maintain the behavior change and to attend the counseling.	
		• How did you experience this aspect of the intervention?	
		• Which factors contributed to the problem?	
		• Which factors helped you to overcome this difficulty?	

#### Occupational physicians, medical assistants, and manager

In order to maximize response, the managers of the two largest OHS were asked for their consent to interview the OPs and medical assistants during their working hours. The OHS managers then arranged for the interviews to be scheduled. The OHS managers were approached directly for an interview. All OHS professionals gave their consent to participate by e-mail.

The OPs, medical assistants and manager were shown a short description of the intervention to introduce the subject of the interview. The respondents were then asked how they currently operated, what the implications of the implementation might be, and how they would respond to these implications. The topic list of the OP interview was focused on facilitators and barriers for performing employee screenings and referring the CVD high risk group to a lifestyle counselor. Medical assistants were interviewed about employee screening. Because the manager had a coordinating role, the topic list touched upon multiple aspects of the implementation process including recruiting and training the lifestyle counselors, instructing the OPs and medical assistants, overseeing OPs and medical assistants who select and refer employees, and planning counseling sessions. The OP interviews lasted one hour on average; the duration ranged from 45–65 minutes. On average, the interviews with the medical assistants lasted 25 minutes; the duration ranged from 15–45 minutes. The interview with the manager lasted about 1.5 hours.

#### Counselors

The counselors who had participated in the earlier trial of the HUC intervention in 2006–2010 were asked to participate. Consent was given by e-mail. Two focus group sessions were organized: one in the south of the Netherlands and one in the north. During the focus groups, two researchers were present. One moderated the focus group, while the other took notes and operated the audio recorder. It had been several years since the counselors had applied the HUC intervention. In order to stimulate recall, the subject of the focus group was introduced with general questions about the earlier trial, such as: "What were useful elements of the intervention?". Then, the counselors were asked about three problematic topics that had arisen during the earlier process evaluation: 1) scheduling 5–7 consultations within a period of six months, 2) applying the motivational interviewing technique, and 3) motivating participants to maintain their behavioral change and attend counseling [18]. The three topics were introduced one after another by reminding the respondents about the results of the earlier process evaluation. For example: "The evaluation of the intervention showed that it was difficult to schedule all consultations within six months". The counselors were asked how they had experienced this aspect of the intervention, which factors had contributed to the problem, and which factors had helped them to overcome this difficulty. Respondents were encouraged to actively participate. Both focus groups lasted about two hours.

### Analysis

The interviews and focus groups were audiotaped and transcribed verbatim. The data was coded using Atlas.ti and open coding with constant comparison [32, 33]. The interviews with the employees and the OPs were coded by two coders (ST and ES). The interviews with the manager, the medical assistants and the counselors rendered less codes, which is why they were coded by one coder (ES). The second coder (ST) read the coded transcripts and checked the codes. The coders had several meetings to reach consensus on codes where discrepancies had arisen. The responses to the open question "Which factors have a role in your decision to sign up for the intervention" were analyzed in the same manner as the reactions to the statements on the cue cards. In a second step, the codes were categorized into broader categories, or themes. A summary was made for each respondent, making the themes more salient. For each respondent, quotes related to each identified theme were entered into a matrix, which made it easier to see the similarities and discrepancies among themes. The themes were then classified according to implementation levels as described by Fleuren, Wiefferink and Paulussen.

## Results

Interviews were held with 20 employees, 8 counselors, 4 OPs, 4 medical assistants, and 1 manager. Table 2 shows the sample size, response rate, and gender per respondent group. The employees who declined to participate in the interview said they lacked the time. The counselors who declined did so because they had changed employer (N = 4), had no time (N = 3), were on sabbatical (N = 1), could not be reached (N = 1), or for unspecified personal reasons (N = 1). One of the two managers could not participate due to prolonged sick leave. There was no manager in a similar position; therefore, no substitute was available to be recruited.

**Table 2:** **Characteristics of the respondents and method of data collection**

Respondent group	Number	Response rate	Male/Female
Employee	20	80%	17/3
Occupational Physician	4	100%	3/1
Medical Assistant	4	100%	0/4
Counselor	8	40%	4/4
Manager	1	50%	1/0

All respondent groups identified both barriers and facilitators to the implementation of the HUC lifestyle intervention. The respondents named themes that were closely related to their role in the implementation process. Employees addressed their individual characteristics, social environment and work. OPs talked about factors that motivated them to make a referral. Counselors named factors that influenced their ability to motivate the employees. The manager named financial implementation aspects and the fit of the intervention with existing procedures. The medical assistants mentioned time pressure, as did the other professionals. The following paragraph gives a more detailed description of the reported barriers and facilitators.

### Barriers and facilitators named by employees

In response to the question whether they would be willing to sign up for the intervention, 11 employees indicated that they would be willing to sign up, 4 were undecided, and 5 were not willing to sign up. Of the three women, two were willing to sign up, and one was undecided. When asked why they were or were not willing to participate, the employees named a combination of themes. Table 3 shows a categorization of these themes by implementation level of the framework of Fleuren, Paulussen and Wiefferink [28]. As can be seen in the table, most themes were related to the implementation level of the target group. One theme was related to the intervention level. Most themes were perceived as a barrier by some employees and as a facilitator by others (cells marked as B/F). Only one theme was perceived as a barrier by all respondents (cells marked as B).

**Table 3:** **Categorization of the themes per respondent group into implementation levels**

Implementation level	Theme	Employee	OP ^1^	Medical assistant	Counsellor	Manager
Socio-political context at the societal level	-	-	-	-	-	-
Characteristics of members of the target group	Risk perception	B^2^/F^3^				
	Preference for independence	B/F				
	Social support and culture	B/F				
	Interference with work	B/F				
Organization and Facilities	Lack of time		B	B	B	B
	Financial incentives					B/F
Health Professional	Fit intervention with perceived task OP		B/F			
	Relationship between OP and other stakeholders		B/F			
	Proficiency in motivational interviewing				B/F	
Intervention	Added value	B/F				
	Fit with existing approach					B/F

^1^Occupational Physician.

^2^Theme was perceived as a barrier.

^3^Theme was perceived as a facilitator.

#### Risk perception

Most employees who were willing to sign up for the intervention first named the hypothetical elevated CVD risk as their reason. Employees who were not willing to participate often reported not feeling at risk. In the employees’ opinion, both the OP’s advice and the PME results contributed to an employee’s risk perception.

Employee A: "I would participate if I really had an elevated risk. See, if the doctor would say that I have an elevated risk, then I would participate".

In addition to this more immediate risk perception, general health awareness appeared to facilitated the employees’ willingness to sign up for the intervention. For example, one employee felt he needed to change his lifestyle, now that he was getting older:

Employee B: "I try, because I see people around me who are over 50 and are pretty huge, who simply have complaints. And that gets you thinking: ‘I don’t want that’. […] My dad also died young, so yeah".

He explained that he had taken up sports recently, not because it gave him pleasure, but as an investment in his health. He raised many practical issues, such as participation costs, travel distance, and the lack of parking facilities. Employees who had been raised with a healthy lifestyle and who found that a healthy lifestyle alone was gratifying were much more optimistic about overcoming practical barriers.

Employees who were willing to participate in the intervention usually indicated that, if they had an elevated CVD risk they would be glad that it had been detected so that they could do something about it. The employees who were not willing to sign up for the intervention were less positive about the possibility of taking preventive measures.

Employee A: "I’ll see about that [signing up] when I actually have CVD; that’ll be soon enough to take action".

The absence of health complaints was often named as a reason for not signing up for the intervention. Even when employees reported having risk factors, such as being overweight or taking medication to lower cholesterol, they expressed feeling healthy enough and considered that their lifestyle was healthy, and thus did not feel that they should be part of the target group. To explain why they believed they did not need to sign up, they emphasized their general health awareness, efforts concerning a healthy lifestyle, and physical activity during work.

#### Preference for independence

Some employees were receptive to the idea of having an elevated CVD risk, but indicated they would prefer to change their lifestyle by themselves. One employee explained that for him, this was really a matter of principle.

Employee C: "I think you should be as independent as possible from doctors and medication. You have to start with yourself".

For these employees, it was not only important to manage their problems on their own, but they were also confident that they would be able to do so. Employees with a strong preference for independence appreciated the fact that the intervention gave room for participants to determine the course of action. However, there were other employees who specifically liked the idea of a health professional telling them what to do.

Employee D: "I think the person that comes to you must take the initiative. See, I may well be thinking: ‘I’m just going to keep living like this’. So it’s very important that the person that comes to you gives you tips, that sort of stuff, and refers you to a dietician, and so on".

#### Added value of the intervention

While it was clear to the employees that the goal of the intervention was to lower the CVD risk, it was unclear what the intervention approach was. Even though the information given in the beginning of the interview explicitly stated that the role of the counselor was to motivate, most employees expected to receive information or medical advice from the counselor. Some concluded that receiving information from the OP or the internet would be sufficient to tackle the lifestyle change by themselves.

Employee C: "If it’s cholesterol, you need more guidance. You can’t monitor that yourself. I mean, if I stand on a weighing scale, I can see if I’m too heavy".

#### Social support and culture

The employees named two people who played a role in their decision to sign up for the intervention: the OP and the employees’ partner. The OP’s advice weighed heavily for almost every employee. The employees did not mention spousal support spontaneously, but their reaction to the statements on the cue cards indicated that their spouse’s approval would influence their decision. On the other hand, the culture in the construction industry seemed to discourage openly addressing health issues. In his reaction to the statement, ‘If I participate, others might think there is something wrong with me’, one employee explained:

Employee A: "If someone says: ‘I have a headache’, you say ‘That’s too bad’. Because, what else can you do about it? I think women talk about this more than men do. Men don’t talk about that stuff".

An employee who had recently begun working in the construction industry observed:

Employee E: "They don’t want to show each other that they’re vulnerable. Everyone is made of steel, nobody has any complaints".

According to this employee, talking about health among employees in construction is simply not done.

#### Interference with work

Interference with work was perceived as a barrier for signing up for the intervention.

Employee F: "And that takes place during working hours? (…) They [the employers] do have a problem with that at the moment. (..) The houses don’t sell, so that has a whole lot of consequences".

One employee indicated that he considered participation in a lifestyle intervention to be sensitive information that he would rather keep from his employer.

Employee G: "Especially nowadays, where you get sacked for the smallest things. (…) Your employer will know you belong to a risk group that might cost him money".

However, most employees either stated that their employer’s opinion had no effect on their decision to sign up for the intervention, or that they expected their employer to support their decision to improve their lifestyle.

### Barriers and facilitators named by professionals

The professionals addressed issues that were closely related to their position in the implementation process. The OPs and counselors addressed issues related to the health professional, while the manager and the medical assistants generally addressed the organizational level.

#### Lack of time

All professionals cited a lack of time as a barrier to implementation. The counselors explained that having enough time for the first consultation was necessary in order to build trust in their relationship with the employee. For the OPs, medical assistants, and manager, one barrier was the lack of remuneration for the screening and referral process. It was left to the OHS to decide whether or not to invest or increase the professionals’ workload. Some OPs worked with a commercial target, for which the promotion of a healthy lifestyle was not relevant. Any measure to make implementation of the intervention cost as little time as possible was therefore seen as helpful.

OP 1: "By not making it difficult for the OPs; by not making them do lots of additional things. Because that makes them cranky. But if there’s a good product, and it’s easily accessible, and they can refer the employee easily, and there’s a folder available, and there’s [information on] the internet, you know, maybe that’s a bad reason, but if you make it easy for them…"

According to the OPs, time pressure makes it necessary for them to prioritize. The large number of competing issues on an OP’s agenda make it a matter of personal motivation for whether or not they would invest their time in lifestyle management.

#### Fit Intervention with perceived task occupational physicians

One of the factors that contributed to the OPs’ motivation to refer an employee was the intervention fit with what the OPs perceived as their main task. OPs thought differently about whether CVD risk management was the OP’s task. One thought that OPs working on CVD risk management meant encroaching on a GP’s task, while another thought that prevention and lifestyle were becoming increasingly important in occupational health care.

OP 1:"Traditionally, the PME was all about: "Is your back straight, are your knees well", physical examination, you know, very medical. Nowadays, it’s more about lifestyle and whether you can keep working till you’re 67. I think many OPs still focus on the technical aspects. While a carpenter might well be able to keep working till he’s 67, even if his back isn’t straight. And I don’t know if everybody is keeping up with this development".

#### Relationship between occupational physicians and other stakeholders

The overlap of the OP’s tasks with the GPs’ tasks also presented more practical problems. One OP explained that he was careful not to make unnecessary referrals to GPs.

OP 2: "I’ve seen it more than once. You tell someone: ‘You have a high cholesterol level’, and people come back: ‘My GP thinks that’s nonsense’. […] As an OP, you have to watch out, because you have to keep working with this GP in the future".

A clear division of tasks and following GP guidelines were seen as ways to preserve a good working relationship with the GP.

One OP admitted that negative experiences with lifestyle counselors had become a considerable barrier for making a referral.

OP 3: "At a certain point I was like: ‘Whatever, I’m not doing this anymore. It’s terribly expensive, and if you guys are only going to complain that clients are not motivated, this is not going to work’. So I stopped referring internally. I thought: good for the GP".

One OP explained that his willingness to make a referral depended on the commitment he felt to a particular company and its employees.

OP 1: "And then there are the small companies […] with maybe five painters, three tilers, and then you don’t know the company that well, you immediately feel less committed, and you’ll work less hard for them".

One OP also talked about how he would like to have more continuity in his contact with individual employees. He suggested progress reports from the counselors, or follow-up phone calls with the employees.

A lack of motivation among employees was a frequently mentioned barrier for OPs. Given the time pressure OPs experience, some were reluctant to invest their time in promoting of a healthy lifestyle if there was a real chance that the employee was not interested.

OP 2: "You make choices according to what someone wants, and where you think you can make a difference. I really think a healthy diet is extremely important. With those people, I don’t even bring it up. Because you can tell just by mentioning it: ‘No, we’re doing well, I don’t need to know any more about this’ ".

To tackle the lack of employee motivation, OPs felt that training in motivational interviewing would be a useful, if not necessary tool. Both OPs and counselors thought that their efforts to motivate the employees would be facilitated by setting the right expectations. For example, the OPs recommended hanging up posters about lifestyle in their waiting rooms, and the counselors wanted the OPs to inform the employees during their PME about what to expect from the counseling.

#### Proficiency in motivational interviewing

For the counselors, the central issue for implementation was their mastery of the counseling technique. To improve their motivational interviewing skills, they needed sufficient practice. They recommended organizing the motivational interviewing training in small groups, so that each counselor had the opportunity to actively participate during role play. After the training, a steady flow of referrals was deemed essential to maintain and further improve their skills. The consultation setting also made a difference according to the counselors. The intervention included both face-to-face and phone consultations. According to one counselor, the phone consultations were not the ideal setting for motivational interviewing.

Counselor: "Checking the progress was the most you could achieve during a phone consultation. But not to increase motivation or deal with real problems".

#### The fit with the current approach

For the manager, implementation of the intervention greatly depended on the fit with the OHS’ current approach. Any adaptation of the status quo meant a certain investment in terms of working hours. Implementation was seen as less problematic if there was a fit with national law, rules and regulations of the construction industry, the OHS’ vision and current working procedures, the professionals’ task description and capacity building plan. The fact that only employees aged 40 years and over were eligible for the intervention posed a problem for the manager.

Manager: "If someone under 40 takes sick leave because of CVD, how long will he be absent, and if he comes back, how much is this going to cost the industry and the employer? This is not what we call prevention. I even think this is age discrimination".

#### Financial incentives

Another issue that was crucial for the implementation from the manager’s point of view was financial incentives. For example, there was no remuneration for the recruitment activities, which made it necessary for the OHS to make an investment.

Manager: "Because somebody has to call this man. Probably several times, because he can’t get a hold of him. Then an appointment has to be scheduled. Then a letter has to be sent. It’s not just a matter of one call. This has to count as the first intervention you can put on the invoice, because otherwise the counselor is not going to do it".

On the other hand, the fact that the counseling sessions involved a comparably high number of working hours for the lifestyle counselors was an incentive for the manager to invest in the implementation.

## Discussion

The aim of the present study was to identify barriers and facilitators to the implementation of a lifestyle intervention in the construction industry. The results point to several ways to improve the intervention and the implementation strategy.

An important barrier for employees to sign up for the intervention was a low level of perceived CVD risk. Some employees reported they were physically active and felt healthy, which made it hard for them to imagine having an elevated CVD risk. But even employees who reported having CVD risk factors, such as being overweight or having high blood pressure tended to evaluate their lifestyle as healthy, and they were not compelled to take action until they developed actual health complaints. This is in line with literature on the Protection Motivation Theory, which found that an elevated risk promotes health adaptive behavior [34–36]. It is also in line with research in an occupational setting, where an elevated risk perception was associated with a high perceived need for and a high level of compliance with a mindfulness intervention program in the workplace [37]. There are several factors that explain a low level of risk perception. First of all, some employees might misinterpret signs of CVD risk. A lack of knowledge and misconceptions about their health risk and an adequate response are frequently cited barriers for people’s the uptake of lifestyle changes [21, 26, 38]. Second, research has shown that people underestimate their susceptibility to those risks that can be modified by their behavior, such as lifestyle, as opposed to risks attributed to non-modifiable factors, such as hereditary factors [39]. People are overly optimistic about their own actions and psychological attributes that contribute to a risk. Third, the concept of an ‘elevated risk’ might be too abstract to impact an employee’s risk perception. A qualitative study on risk communication in primary prevention found that patients’ understanding of risk was often unrealistic, dichotomous, and based on personal experience [38]. A study among Dutch construction workers reported that workers found it hard to attach meaning to the concept ‘elevated risk’ [40]. Employees with a low level of education had difficulty distinguishing between an elevated risk and an illness. These results suggest that within the risk communication used in the HUC intervention, behavioral risk factors might deserve more attention. A dual strategy of clearly communicating the comparative risk percentage on one hand, and providing employees with insight into their behavioral risk factors on the other might impact a larger part of the target group.

The target group for the current study seemed to be more heterogeneous regarding their attitude towards counseling than previously anticipated. On the one hand, there was a group of employees who were not motivated to sign up for the intervention because of a strong preference for independence. This finding is similar to what Tod et al. describe as a culture of self-reliance that values ‘strength and the ability to cope and maintain independence’ [41]. On the other hand, there was a group of respondents who saw the added value of the intervention precisely as ‘an expert telling them what to do’. Thus, a one-size-fits-all approach will not suffice for an intervention and recruitment strategy that seeks to reach all subgroups. As Murray et al. suggested, a more tailored approach that offers ‘supported change’ as well as ‘self-managed change’ seems more promising [42].

The added value of the intervention was unclear to the employees. It appeared that employees found it hard to picture counseling on physical activity and nutrition, which is in line with findings of an earlier study [31]. Research has shown that people are more likely to adopt a certain health behavior if they believe it will be effective [28, 34, 35]. In order to reach the target group, the added value of the intervention needs to be communicated more clearly. However, selling the intervention to the target group might also be a problem of gender incongruence. The culture of the construction industry seems to reward the projection of the image of strong and independent men, which might contrast with the soft factor of ‘motivation’ that this intervention targets.

The present study is in line with the literature on factors that facilitate or hamper a referral, replicating the influence of lack of time [21, 24, 43, 44], fit with the perceived task [21, 24], and the physician’s relationship with other stakeholders [21, 24, 45]. Although a lack of time is identified as a barrier in studies from all over the world, part of the explanation for this study’s results may lie in the Dutch context [23, 43, 44]. Occupational health care in the Netherlands was privatized in 1998, and competition has increased further since 2005, when it was determined that Dutch companies were no longer legally obliged to hire OHS. In a study on the implementation of an occupational health guideline to prevent weight gain, OPs indicated that implementation depended on whether they were given extra time for the implementation, and whether the implementation would be at the expense of other tasks that were stipulated in their contract [46]. The OPs’ struggle to balance quality and efficiency demands is manifested in the present study. OPs indicate that time pressure makes it necessary to make choices, which explains why the OP’s motivation takes such a central place in the present study. Not only should an intervention be time efficient and flexible to maximize the chances of implementation, it should be rewarding for the professional who implements it. The results point to several ways how to make referring to the lifestyle intervention more rewarding.

First, OPs expressed frustration about a lack of motivation to alter their lifestyle among many employees. Physicians experience the lack of motivation among patients as an even more important barrier to referrals than the lack of time [45]. Laws et al. observed that physicians are more likely to take action when they set their goals in terms of the process of change rather than in terms of behavioral targets, and when they evaluate the results of their work at the population level rather than the individual level [24]. In addition to a shift from outcomes to process, the literature suggests there is a motivating effect of observable results [47]. OPs in the present study confirm they need observable intervention results. Progress reports from the lifestyle counselor could provide the OP with tangible results, help to form a frame of reference for what can be achieved, and shape the perception that a referral is part of a concerted effort.

OPs indicated that a collaborative relationship with counselors would facilitate referral. An earlier study showed a general preference for physicians to refer to in-house services, although this was attributed to better accessibility and the patient being more familiar with the location [23, 48]. In the present study, it seemed to be an issue of trust in the competence of the other professional. There is evidence that a referral depends on the extent to which OPs believe in the effectiveness of the intervention [22]. Our study took place in two large organizations with offices spread over the whole country, thus the OPs and lifestyle counselors rarely met in person. For OPs and lifestyle counselors to meet each other is a first step for establishing a working relationship based on trust. Reporting back on the results of the counseling trajectory can further help to establish knowledge-based trust in the competence of the lifestyle counselor. Proactive promotion of the counselor’s services among OPs has been shown to convince OPs who do not know what counselors have to offer [49].

There was no consensus among OPs on whether or not OPs had a legitimate role in the promotion of a healthy lifestyle. Other studies indicate a positive effect of role congruence on referral [22]. OPs might benefit from clear policy statements and OHS management targets that integrate the promotion of a healthy lifestyle within their organization. Guideline developers should clarify if and how consultation and collaboration with an employee’s GP is advisable. They should strive for coherence with existing GP guidelines and consider the adverse effects that the implementation of a guideline could have on the relationship between the OP and GP. Decision makers within OHS and OP interest groups need to employ strategies on how to implement those guidelines.

In sum, OPs need to be motivated to invest their time in the lifestyle agenda by making a referral personally and professionally rewarding. Evaluations, targets, progress reports, and internal guidelines should all contribute to the OP’s perception that investing in lifestyle promotion is instrumental to reaching his professional goals.

### Implementation levels

The implementation levels of the model by Fleuren et al. were used as a starting point for structuring the results of this study [28]. During the course of the analyses, we found it confusing that the category ‘socio-political level’ includes factors that are located at the societal level, as well as at the individual level. We therefore adapted the model by splitting the category ‘socio-political level’ into ‘socio-political context at the societal level’ and ‘characteristics of members of the target group’ (see Table 3). Respondents generally named factors related to their own role in the implementation process. In general, employees named themes at target group level, the OPs and counselors named themes related to the health professional level, and the manager and medical assistants named themes that were related to the organizational level. This result was expected, since respondents were instructed to talk about their individual role in the implementation process, and not the implementation process as a whole. This might explain why there was little overlap in themes among respondent groups. Lack of time was the only factor named by multiple respondent groups.

### Methodological issues

The present study has several strengths. First, it describes barriers and facilitators from the perspective of a range of stakeholders, which leads to a broad overview of the relevant issues. In particular, the interviews with the employees provided new insights. To our knowledge, there are few qualitative studies of barriers and facilitators to signing up for an intervention [42]. Also, studies on signing up for interventions in an occupational setting are rare [50].

This study also has limitations. The interviews were held before implementation with the aim to use the results to optimize the implementation strategies prior to the start of the actual implementation. This meant that the OPs, medical assistants and manager had no firsthand experience with and only limited information about the intervention. Additional interviews after implementation might reveal barriers and facilitators that the respondents were unaware of before the implementation.

Distinct methods of data collection were used for the counselors and the other respondent groups, which limits comparability of the results. This limitation should be taken into account when interpreting the results.

For some groups (assistants, counselors), saturation was reached, after which no further interviews took place. For other groups (OPs, employees, manager), the number of participants was too small and did not result in saturation. The interviews were performed during the six months prior to actual implementation. Once implementation had started, no further interviews could be performed, because the results would not have been comparable to the results generated before implementation. Nevertheless, with the available data, the present study offers an overview of the most salient factors that influence the implementation process.

All professionals who participated in the interviews knew they might be involved in the implementation of the HUC intervention and this might have influenced their responses during the interview.

All respondents were recruited through the two largest occupational health providers in the Netherlands. Small service providers were not represented in the sample, meaning that the results may not reflect issues that arise in small OHS.

The intervention targets individuals with an elevated CVD risk. However, the sample of employees was not selected based on CVD risk. This might have resulted in a sample that is more heterogeneous regarding CVD risk and lifestyle than the target group. Furthermore, the employees’ response e might differ from respondents who are confronted with an actual elevated CVD risk. Literature indicates that perceived susceptibility and perceived severity of a threat influences the intention to adopt protective behavior, although the influence is smaller and less consistent than that of the perceived response efficacy and perceived self-efficacy regarding the offered protective measure [34–36].

The counselors had participated in the earlier trial in 2007. In the present study, the counselor were interviewed about their experiences with the intervention that had occurred five years prior to the interview, which means they might have been prone to recall bias.

## Conclusion

Employees and professionals named a combination of factors that they thought would hamper or facilitate the future implementation of the intervention. In relation to the willingness to sign up for the intervention, employees named risk perception, social support, the added value of the intervention, preference for independence, and interference with work. All professionals named lack of time as a barrier to implementation. OPs named the intervention’s fit with their perceived task, and their relationship with other stakeholders. Lifestyle counselors addressed several ways to improve their proficiency with the counseling technique. The manager addressed the intervention’s fit with the OHS’ current approach and financial incentives. The main conclusions that were drawn from this study were: 1) the implementation strategy needs to be tailored to the various subgroups within the target group, and 2) OPs can be motivated to invest their time in the lifestyle agenda by making a referral personally and professionally rewarding. The results of the present study provide concrete ways to adapt the HUC intervention and the implementation strategy in the Dutch construction industry.

## Electronic supplementary material

Additional file 1: **Title of data: Statements presented to employees during interviews.** Description of data: Statements that were presented to the employees on cue cards during the interviews as a possible barrier or facilitator for them signing up for the intervention. (PDF 13 KB)
