# Supplementary material for: A qualitative study of the anticipated barriers and facilitators to the implementation of a lifestyle intervention in the dutch construction industry
Source: BMC Public Health. 2014 Dec 23;14:1317. doi: 10.1186/1471-2458-14-1317 (PMC4326182; doi:10.1186/1471-2458-14-1317)
Supplement: Supplementary file 1 — Additional file 1: Title of data: Statements presented to employees during interviews. Description of data: Statements that were presented to the employees on cue cards during the interviews as a possible barrier or facilitator for them signing up for the intervention. (PDF 13 KB) [file 12889_2014_7481_MOESM1_ESM.pdf]

Additional File 1: Statements presented to employees during interviews

My health is none of Arbouw's concern.
I'm entitled to this treatment.
My wife thinks it's important I participate.
I know somebody with cardiovascular disease.
Nobody else at work does anything about it either.
On the construction site, all you can get is French fries anyway.
If I sign up, I'll worry about my employer finding out.
If I sign up, people will think there's something wrong with me.
My employer thinks it's important I participate.
It's no use; I cannot get myself to do it.
It's no use; I won't be able to keep it up.
I'm not a big talker.
I want to keep working a little longer.
After work, I simply don't have the energy.
My health complaints are bothering me.
Six months; that's way too long.
Forty-five minutes; I cannot spare that much time.
I simply enjoy good food.
I'm not a sports person.
If I sign up, I'll have to drive all the way over there.
It won't cost me any working time.
After all, it's free of charge.
This is a course where it's up to me what I do or what I don't do.
I get so much exercise anyway.
I have an elevated risk, so I have to do something about it.
The doctor says it's necessary for me to do this.
